# Cathodic electrodeposition of amorphous tungsten oxide dihydrate film for dual-band electrochromic modulation†

**DOI:** 10.1039/d4ra08851d

**Published:** 2025-02-18

**Authors:** Qiyuan Zhang, Ruoming Du, Aihua Yao

**Affiliations:** a School of Materials Science and Engineering, Tongji University Shanghai 200092 China 07182@tongji.edu.cn; b Key Laboratory of Advanced Civil Engineering Materials, Ministry of Education, Tongji University Shanghai 200092 China

## Abstract

Recent advances in hydrated tungsten oxides (WO_3_·*n*H_2_O, *n* = 1, 2) have highlighted their potential for dual-band electrochromic applications. However, achieving both high optical modulation and robust cycling stability remains a challenge. This study presents a cost-effective and straightforward cathodic electrodeposition method to fabricate amorphous tungsten oxide dihydrate (WO_3_·2H_2_O) films using a precursor solution primarily composed of monomeric diperoxotungstate. The open framework and interlayer structural water in WO_3_·2H_2_O enable exceptional dual-band electrochromic performance at super-low switching potentials (−0.5 V for full coloration and 0.5 V for full bleaching) in a 1.0 M LiClO_4_/propylene carbonate (LiClO_4_/PC) electrolyte. Key features include high optical modulation (∼92% at 633 nm and ∼86% at 1100 nm), fast response times (17.5 s for bleaching and 18.1 s for coloration at 633 nm; 5.0 s for bleaching and 7.1 s for coloration at 1100 nm), high coloration efficiencies (204.2 cm^2^ per C at 1100 nm and 72.3 cm^2^ per C at 633 nm), and exceptional cycling stability (retaining 94% of initial optical modulation after 2000 cycles and 76% after 10 000 cycles at 633 nm). The interlayer water in WO_3_·2H_2_O plays a critical role in facilitating pseudocapacitive Li^+^ intercalation, enabling control over optical properties across the visible and near-infrared (NIR) regions while maintaining structural integrity. Additionally, the scalability of the method was demonstrated through the successful fabrication of large-area films (10 cm × 10 cm) and prototype electrochromic devices.

## Abstract 2

Amorphous WO_3_·2H_2_O films were electrodeposited from a monomeric diperoxotungstate solution derived from [WO_2_(O_2_)H_2_O]·1.66H_2_O powder. The open framework and interlayer water in WO_3_·2H_2_O enable outstanding dual-band electrochromic performance.

## Graphical abstract

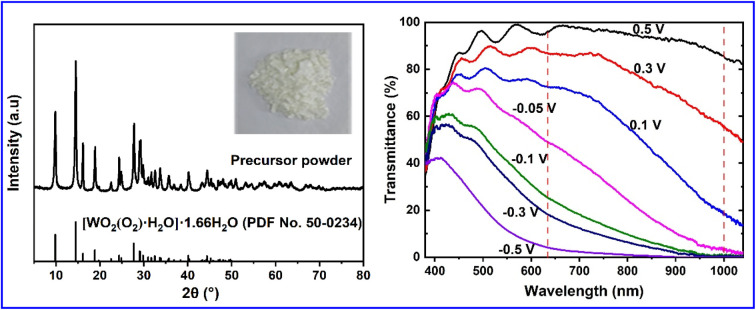

## Introduction

Electrochromism is a phenomenon in which the optical properties – such as transmittance, reflectance, and absorption – of certain electroactive materials undergo stable and reversible changes under an applied electrical potential. This allows for reversible and persistent changes in color or transparency. Over the past few decades, electrochromic materials have gained considerable attention in both academic research and commercial applications. They are particularly valued in technologies such as smart windows, self-dimming rear view mirrors for automobiles, information displays, and energy storage devices, *etc*.^1–3^

With the advancement of electrochromic technology, numerous novel electrochromic materials and devices have been extensively studied. Among them, tungsten trioxide (WO_3_) has emerged as one of the most commercially significant electrochromic oxides due to its large optical modulation, high coloration efficiency, and natural abundance.^4,5^ However, WO_3_ faces challenges such as sluggish ion diffusion kinetics, limited ion storage capacity, and inadequate cyclic stability.^6,7^ Recently, strategies involving interlayer water have been employed to enhance ion diffusion and improve the overall electrochromic performance.^7–10^ In this context, layered tungsten oxide hydrates, WO_3_·*n*H_2_O (where *n* = 1, 2), have attracted increasing attention due to their superior electrochromic and electrochemical properties. These hydrates consist of layers of corner-sharing WO_6_ octahedra with stoichiometric amounts of primary and secondary bound structural water. For example, dihydrate (WO_3_·2H_2_O) contains two types of structural water molecules: one directly coordinated to W^6+^, forming WO_5_(OH)_2_ sheets *via* corner-sharing, and another located between the layers, bound to the lattice through hydrogen bonding.^8,11^ The structural water was found to expand the interlayer spacing, significantly reducing the energy barriers for the ion diffusion, and enhancing ion flux in the WO_3_ framework.^8,9^ J. B. Mitchell^12^ further demonstrated that the confined interlayer water in WO_3_·*n*H_2_O plays a critical role in minimizing local mechanical deformation and stabilizing the structure. This facilitates rapid charge transfer kinetics and effective stress relaxation during electrochemical ion intercalation and deintercalation processes. Moreover, introducing interlayer water enhances local surface plasmon resonance (LSPR), enabling a transition from battery-like to pseudocapacitor-like behavior.^8,11^ As a result, hydrated WO_3_ has emerged as a promising NIR and dual-band electrochromic material, allowing independent control over NIR and visible light transmittance. For example, W. Zhao *et al.*^9^ fabricated amorphous tungsten oxide hydrates film by drop casting a solution of WCl_6_ in isopropyl alcohol. It was confirmed that the WO_3_·0.9H_2_O film exhibited superior NIR electrochromic performance compared to species with lower interlayer water content (WO_3_·0.5H_2_O and WO_3_·0.1H_2_O). They found that the enhanced NIR electrochromic capability was associated with the dominant pseudocapacitive behavior promoted by the structural water. Fortunato *et al.*^13^ demonstrated that crystalline WO_3_·H_2_O achieved a dual-band electrochromic response, driven by polaron absorption in the visible region and plasmon effects in the NIR region, depending on the amount of Li^+^ injected into the material. However, the modulation in the visible and NIR regions was limited to 13% and 59%, respectively. More recently, Sun X. *et al.*^14^ developed Ti-doped WO_3_·2H_2_O nanosheet films with enhanced dual-band electrochromic performance, achieving optical modulation of 83.8% at 633 nm and 72.5% at 1050 nm. However, their cyclic stability remains unsatisfactory, thus highlighting the need for further improvements.

WO_3_ hydrates are typically synthesized through liquid-phase synthesis routes such as hydrothermal, sol–gel and electrodeposition processes. Compared to more complex and highly expensive high-temperature and/or high-vacuum methods like sputtering and chemical vapor deposition (CVD), liquid-phase synthesis offers several advantages, including low cost, mild reaction conditions, easy control over film thickness and morphology, and scalability for large-area production.^15^ These processes usually start with an acidified precursor, H_2_WO_4_, obtained by acidifying an aqueous sodium tungstate (Na_2_WO_4_) solution with a strong acid (*e.g.*, HCl or HNO_3_).^8,10,12,16^ In electrodeposition process, this acidic precursor reacts with H_2_O_2_ to form a peroxotungstic acid solution, which serves as the electrolyte for WO_3_ film deposition.^12,13,17^ However, the acidified precursor obviously contains contaminating cations like Na^+^, which can adversely affect the performance of final product.

In this study, we proposed a cost-effective and straightforward cathodic electrodeposition method to fabricate amorphous tungsten oxide dihydrate (WO_3_·2H_2_O) films. The process employs an impurity-free precursor solution primarily composed of monomeric diperoxotungstate, prepared by dissolving a precursor powder with the composition [WO_2_(O_2_)H_2_O]·1.66H_2_O. The low degree of polymerization of this precursor provided enhanced stability compared to commonly reported dimeric tetraperoxo species, thereby improving the reliability and efficiency of the electrodeposition process. Our results demonstrate that the amorphous WO_3_·2H_2_O films fabricated using this method exhibit outstanding electrochromic performance, including a broad spectral response, significant optical modulation, fast switching speed, and excellent cycling durability. Additionally, the feasibility and scalability of the proposed method were validated through the successful fabrication of large area films (10 cm × 10 cm).

## Experimental

### Materials and methods

#### Materials

Tungsten metal powder (particle size 1–5 μm, 99.98% purity) was purchased from Aladdin Reagent Co., Ltd. Isopropyl alcohol (AR) was purchased from Shanghai Titan Technology Co., Ltd. Hydrogen peroxide (30%, GR), lithium perchlorate (LiClO_4_, AR), lithium chloride (LiCl, AR), acetone (AR), and absolute ethanol (AR) were obtained from Sinopharm Group Chemical Reagent Co., Ltd. Indium tin oxide (ITO)-coated glass slides, with dimensions of 2 cm × 5 cm and 10 cm × 10 cm, and a square surface resistivity of 7–10 Ω sq^−1^, were purchased from Luoyang Tengjing Glass Co., Ltd.

#### Preparation of precursor solution

Under optimized conditions, 4.60 g of tungsten powder was placed in a 500 mL beaker, and a mixture of 25 mL H_2_O_2_ (30%) and 30 mL isopropanol was quickly poured into the beaker. Following a vigorous exothermic reaction, the product was transferred to an incubator for evaporation at 60 °C until dry, yielding a white powder. Next, 7.40 g of the powder was dissolved in a mixture of deionized water and isopropanol (volume ratio 7 : 3), and 1 mL of H_2_O_2_ (30%) was added as a stabilizer. The solution was stirred continuously for 36 h at 25 °C, resulting in a transparent solution with a pH of 1.8. The total tungsten content in the precursor solution was determined by inductively coupled plasma optical emission spectroscopy (ICP-OES, PerkinElmer Optima 8300, USA), while the peroxide concentration (free H_2_O_2_ + W-bound O_2_^2−^) was measured *via* classical manganometric titration.^18^

#### Electrodeposition of amorphous WO_3_·2H_2_O film

Prior to electrodeposition, ITO-coated glass slides were cleaned sequentially with ethanol, acetone, and deionized water in an ultrasonic bath for 10 min. Electrodeposition was conducted using a CHI660D electrochemical workstation (Shanghai CH Instruments, China) in a conventional three-electrode setup, with ITO glass slide as the working electrode, a Pt plate (5 cm × 5 cm for 2 cm × 5 cm films and 12 cm × 12 cm for 10 cm × 10 cm films) as the counter electrode, and an Ag/AgCl (3 mol L^−1^ KCl) as the reference electrode. Potentiostatic electrodeposition was performed by applying −0.5 V *versus* the reference electrode for 30 min, resulting in the formation of a dark-blue film on the ITO substrate. The selection of −0.5 V as the deposition potential is based on repeated experimental trials. We found that the film electrodeposited at −0.5 V to −0.6 V exhibited the best electrochromic performance. Films deposited outside this potential range, either lower or higher, showed poor cycling stability. The film was then rinsed thoroughly with deionized water and dried at 25 °C in an air stream. After 12 h of exposure to ambient air, the film fully bleached.

#### Characterization

The phase composition of the precursor powder and electrodeposited film was analyzed using X-ray diffraction (XRD, Rigaku D/max2550) with Cu Kα radiation at 40 kV and 40 mA. The microstructure of the film was examined using a Laser Confocal Raman Microscope (LabRAM HR Evolution) with a 514 nm laser excitation. The chemical composition and oxidation states were investigated by X-ray photoelectron spectroscopy (XPS, Escalab250Xi, Thermo Scientific) with a 500 μm diameter beam of monochromatic Al Kα radiation. The surface morphology was observed using a field-emission scanning electron microscope (FESEM, Hitachi S-2360). The thickness of the film was determined using an ellipsometer (M-2000v, J. A. Woollam RC2).

Electrochemical and electrochromic properties were assessed in a three-electrode setup, with the WO_3_·2H_2_O film on ITO as the working electrode, a Pt plate (5 cm × 5 cm for 2 cm × 5 cm films and 12 cm × 12 cm for 10 cm × 10 cm films) as the counter electrode, and an Ag/AgCl (3 mol L^−1^ KCl) as the reference electrode. The electrolyte used was a 1.0 M LiClO_4_ solution in propylene carbonate (LiClO_4_/PC). Electrochemical characterization was performed using cyclic voltammetry (CV), chronoamperometry (CA) and chronocoulometry (CC). All potentials were referenced to an Ag/AgCl electrode. CV curves were obtained by scanning between −0.5 V and 0.5 V at various scan rates. CA measurements were performed by applying constant potential steps (−0.5 V for 30 s, 0.5 V for 30 s) to quantify the response times. CC tests were conducted at applied potentials of −0.5 V and 0.5 V, with each potential held for 30 s. Optical transmittance was measured using a fiber optic spectrometer (Ocean Optics, S2000-VIS) across the wavelength range of 400–1100 nm. Transmittance spectra were recorded at various potentials after allowing the optical signals to stabilize for 30 s, with the transmittance of the ITO substrate subtracted from the results. The cycling stability of the films was evaluated using a double-step CA method, in which the potential was alternated between −0.5 and 0.5 V in a 1.0 M LiClO_4_/PC solution, with a holding time of 30 s at each potential. Optical memory effect of the prototype electrochromic device was evaluated by monitoring the transmittance evolution at 633 nm under open-circuit voltage (OCV) after coloration at −0.7 V.

## Results and discussions

### Preparation and physical characterization

**Fig. 1:**
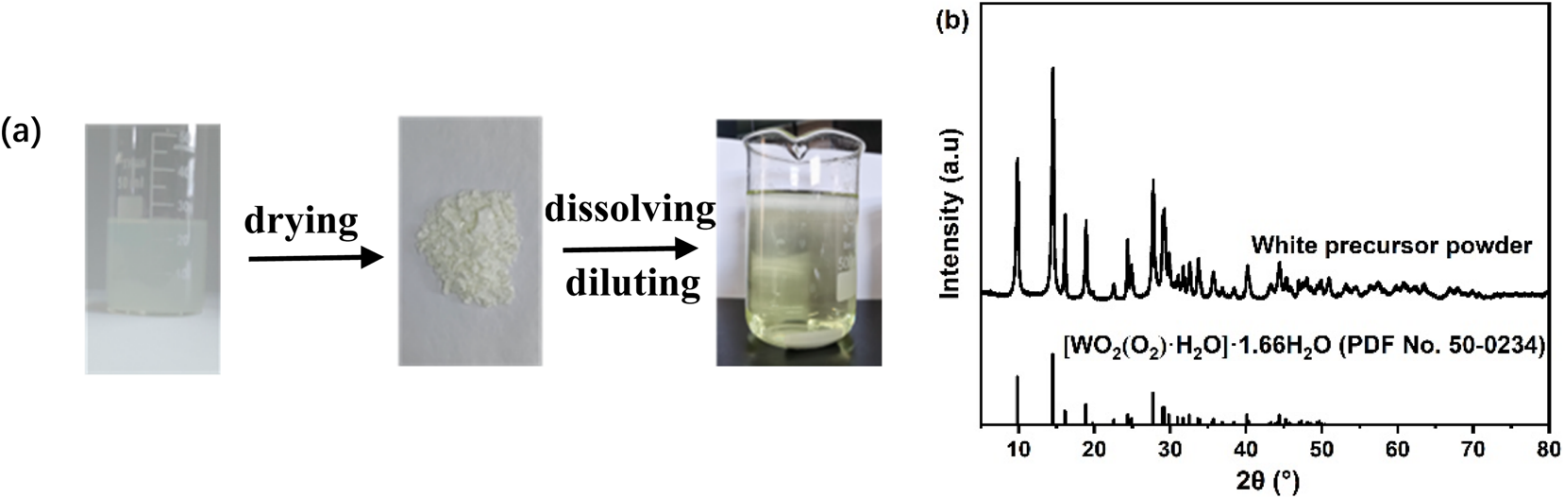
(a) Photographs of the products obtained during the preparation of the precursor solution; (b) XRD pattern of the white precursor powder.

As shown in Fig. 1(a), the reaction between tungsten powder and H_2_O_2_ aqueous solution produces a suspension, which yields white powders upon evaporation at 60 °C. The powders remained stable for at least six months when stored at room temperature in a vacuum container. The XRD pattern of the white powders, presented in Fig. 1(b), closely matches the monoclinic hydrated tungsten peroxide ([WO_2_(O_2_)H_2_O]·1.66H_2_O, JCPDS#50-0234). This is consistent with the findings of Pecquenard *et al.*,^19^ who obtained the same product by slowly evaporating a peroxopolytungstic acid solution. They explained that the product formed through the condensation of the monoperoxo intermediate [WO(OH)_3_(O_2_)(H_2_O)], where the O_2_/W ratio is approximately 1, with (O_2_) representing a peroxide ligand. Based on these findings, we deduce that the predominant form of peroxotungstate in the precursor solution is monomeric diperoxotungstate (O_2_/W ≈ 1), rather than the commonly reported dimeric tetraperoxo species, W_2_O_11_^2−^ (or [(O)W(O_2_)_2_(O)(O_2_)_2_W(O)]^2−^).^18,20^ For the subsequent electrodeposition process, the white powders were dissolved in a mixture of deionized water and isopropanol and the solution was stirred continuously for 36 h at 25 °C, resulting in a pale-yellow solution. To determine the O_2_/W ratio, the concentrations of tungsten and peroxide ligands (free H_2_O_2_ + W-bound O_2_^2−^) in the precursor solution were measured, yielding an O_2_/W ratio of approximately 1.08. This strongly supports our conclusion that the precursor solution primarily consists of monomeric diperoxotungstate. We found that a small amount of H_2_O_2_ was necessary to stabilize the precursor solution, as confirmed by H. Nakajima *et al.*,^21^ who reported that the free H_2_O_2_ prevented the formation of more polymerized species in peroxotungstate solution, ensuring stability. The precursor solution remained stable for at least 7 days when stored at −4 °C.

**Fig. 2:**
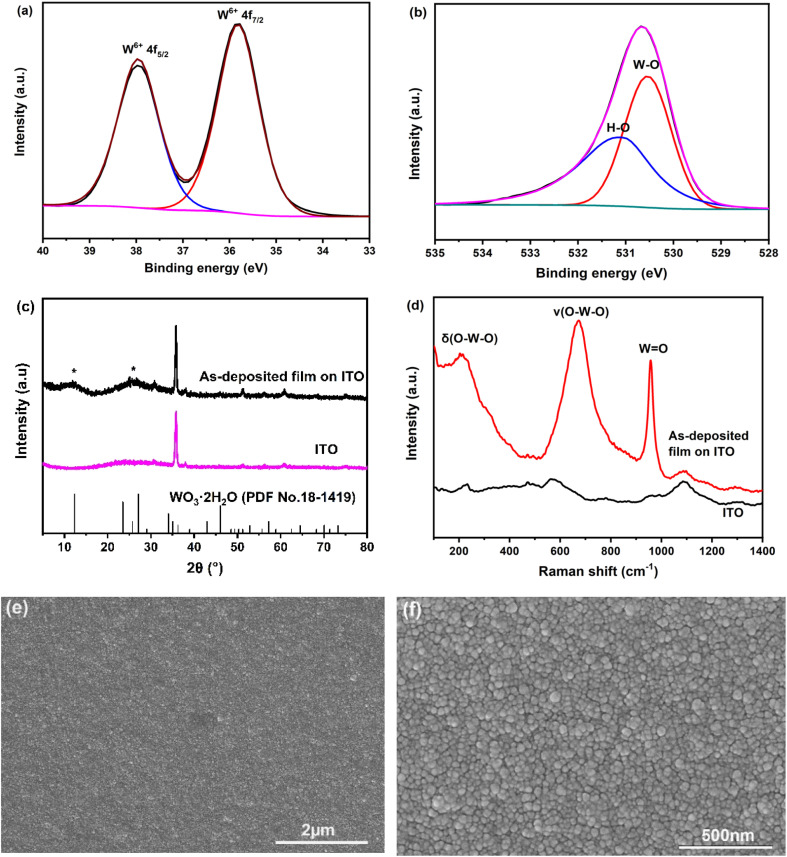
Structural and morphological characterization of the amorphous WO_3_·2H_2_O film electrodeposited on the ITO substrate: (a) and (b) high-resolution W4f and O1s XPS spectra, (c) XRD pattern, (d) Raman spectrum, (e) and (f) SEM images.

The as-deposited film initially appeared dark blue and completely bleached after exposure to ambient air for 12 h. This phenomenon suggests the presence of reduced tungsten, likely in the form of hydrogen tungsten bronze (H_*x*_WO_3_), which forms under the present conditions (pH 1.8 in our case). Upon exposure to air, the reduced tungsten re-oxidizes, leading to the observed bleaching of the film. The thickness of the film electrodeposited using the current conditions was measured using an ellipsometer and found to be approximately 200 nm. XPS was performed to analyze the chemical composition and bonding state of the fully bleached film. The high-resolution W 4f spectrum in Fig. 2(a) displays peaks at 37.7 eV and 35.5 eV, corresponding to W^6+^.^22^ The O1s spectrum in Fig. 2(b) reveals two components: one at 530.5 eV, assigned to W–O bond, and another at 531.5 eV, associated with surface hydroxyl groups and/or coordinated water.^23^ The XRD pattern (Fig. 2(c)) shows, in addition to the characteristic peaks of ITO, two broadened peaks in the low-angle region (2*θ* ∼ 5–30°). These peaks suggest an expansion of interlayer spacing between adjacent WO_5_(H_2_O) octahedral layers due to the presence of structural water. Notably, distinctive diffraction peaks for WO_3_·2H_2_O and WO_3_·H_2_O are typically observed at ∼12° and ∼16°, respectively.^24^ Thus, the broadened peak centered at ∼12.5° confirms the formation of amorphous WO_3_·2H_2_O. The Raman spectra of the as-deposited film, shown in Fig. 2(d), exhibit peaks between 200 and 300 cm^−1^, as well as around 670 cm^−1^, which correspond to the bending [*δ*(O–W^6+^–O)] and stretching [*v*(O–W^6+^–O)] modes of bridging oxygens. The sharp peak at approximately 960 cm^−1^ is attributed to the symmetric stretching mode of terminal W^6+^=O bonds, which is widely used as a spectral marker for amorphous WO_3_·2H_2_O.^25^ The surface morphology of the as-deposited film was observed using SEM. The low-magnification image in Fig. 2(e) shows the formation of a uniform, crack-free film on the ITO substrate. The high-magnification image in Fig. 2(f) reveals that the film is composed of spherical grains with an average size of approximately 40 nm. These nanograins are uniformly distributed across the entire surface.

### Electrochemical and electrochromic performances

**Fig. 3:**
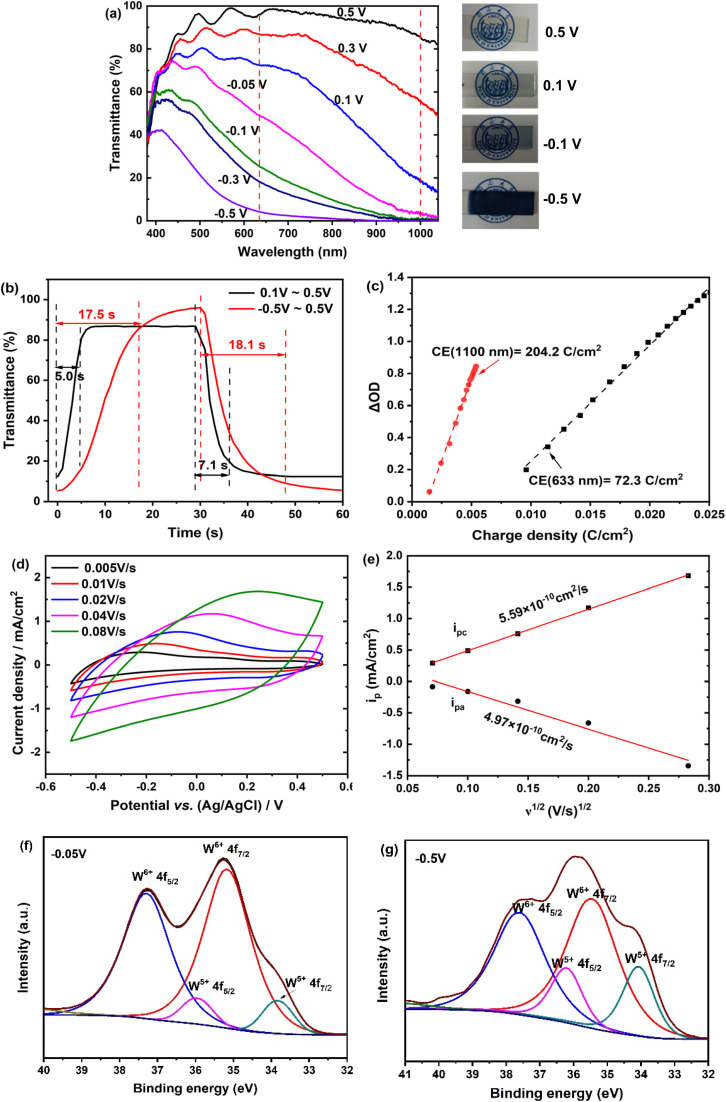
Electrochemical and optical properties of the amorphous WO_3_·2H_2_O film: (a) optical transmittance spectra at various potentials held for 30 s in 1 M LiClO_4_/PC, along with corresponding digital photographs; (b) response times measured at 633 nm and 1100 nm for the films, evaluated by CA steps within the potential ranges of −0.5 to 0.5 V or 0.1 to 0.5 V; (c) variation of optical density (ΔOD) at 633 nm and 1100 nm as a function of charge density, with coloration efficiencies (CE) determined from the slope of the linear region; (d) CV curves at various scan rates; (e) Li^+^ diffusion coefficients calculated from the plots of peak current (*i*_p_) *versus* scan rate (*v*) in (d) using the Randles–Sevcik equation; (f) and (g) high-resolution W4f XPS spectra of the film reduced at −0.05 V and −0.5 V.

The electrochemical and electrochromic performances of the amorphous WO_3_·2H_2_O film were evaluated using a three-electrode setup in a 1.0 M LiClO_4_/PC solution. Optical transmittance spectra were recorded at different applied potentials to determine the optical modulation (Δ*T*) of the film. As shown in Fig. 3(a), the film demonstrates selective modulation of both visible and NIR light transmittance across the wavelength range of 400 nm to 1100 nm. When the applied potential is generally reduced from 0.5 V to 0.1 V, the NIR transmittance decreases significantly, achieving a Δ*T* of 74% at 1100 nm, while the change in visible light transmittance is comparatively small, with a Δ*T* of 18% at 633 nm. At a negative potential of −0.05 V, the NIR transmittance decreases further, reaching a maximal Δ*T* of 86% at 1100 nm, while visible light transmittance remains above 55%. When the potential is further reduced to −0.1 V, the visible light transmittance drops sharply, eventually reaching 5% at 633 nm at −0.5 V, resulting in a maximal Δ*T* of ∼92% at 633 nm. Photographs of the film at various potentials confirm the visible changes, with the film transitioning from transparent at 0.5 V to light blue at −0.05 V, and finally to dark blue at −0.5 V.

The switching response time, defined as the time required to reach 90% of the full modulation, was determined by recording transmittance changes at 633 nm and 1100 nm during potential steps (−0.5 V or 0.1 V for 30 s, followed by 0.5 V for 30 s). From the transmittance–time curves shown in Fig. 3(b), the coloration time (*t*_c_) and bleaching time (*t*_b_) at 633 nm were measured as 18.1 s and 17.5 s, respectively. At 1100 nm, the *t*_c_ and *t*_b_ were much faster, at 7.1 s and 5.0 s, respectively. Another important performance parameter for electrochromic materials, coloration efficiency (CE), was calculated as the change in optical density (ΔOD = log(*T*_b_*/T*_c_)) per unit of injected charge. The slope of the linear portion of the plots in Fig. 3(c) yielded a CE value of 204.2 cm^2^ per C at 1100 nm, which is nearly three times higher than the CE at 633 nm (72.3 cm^2^ per C). The significant difference emphasizes the superior dual-band electrochromic performance of the amorphous WO_3_·2H_2_O film.

To further investigate the electrochromic mechanism, CV profiles of the film were measured at varied scan rates. As observed in Fig. 3(d), the CV curves exhibit a quasi-rectangular shape with broad redox peaks, indicating a typical pseudocapacitive intercalation process. In this process, Li^+^ ions are reversibly inserted into and extracted out of the interlayer space of the WO_3_·2H_2_O structure, enabling a reversible and fast redox reaction. The presence of structural water molecules increases the interlayer spacing, which facilitates faster Li^+^ ion diffusion during charge and discharge processes. This enhanced ion mobility significantly contributes to the observed pseudocapacitive behavior.^26^ The Li^+^ diffusion coefficients, calculated from the plots of peak current (*i*_p_) *versus* scan rate (*v*) (Fig. 3(e)) using the Randles–Sevcik equation,^27^ are 4.97 × 10^−10^ cm^2^ s^−1^ for insertion and 5.59 × 10^−10^ cm^2^ s^−1^ for extraction. These values are higher than those for anhydrous WO_3_ (ref. 27) and commonly studied WO_*x*_ films,^28,29^ and are comparable to those of W_18_O_49_ NWs/Ti_3_C_2_T_*x*_ composite film^29^ and WO_3_·2H_2_O nanoplate film^8^.

XPS analysis of the film reduced at −0.05 V and −0.5 V (Fig. 3(f) and (g)) shows two pairs of characteristic peaks in the high-resolution W4f spectra. Binding energies at 35.5 and 37.6 eV correspond to W^6+^, while those at 34.0 and 36.2 eV are attributed to W^5+^. These results demonstrate that the electrochromic behavior of the amorphous WO_3_·2H_2_O film arises from the redox transition between W^6+^ and W^5+^, driven by the simultaneous insertion and extraction of electrons and Li^+^ ions (WO_3_·2H_2_O + Li^+^ + *x*e^−^ → Li_*x*_WO_3_·2H_2_O). The calculated W^5+^ ratios for films reduced at −0.05 V and −0.5 V were 7.2% and 29.4%, respectively, indicating greater Li^+^ intercalation at lower reduction potentials. These results demonstrate that the amorphous WO_3_·2H_2_O film achieves optical modulation in both visible and NIR regions through varying degrees of charge injection. At lower reduction potentials, limited Li^+^ ions insertion leads to the partial reduction of tungsten to W^5+^, generating additional free electrons that induce plasmonic effects, primarily modulating light in the NIR region.^30^ At higher reduction potentials, increased Li^+^ insertion causes a more significant reduction of tungsten, generating polarons that absorb light in the visible region, thereby enhancing visible light modulation.^13^

### Long-term cycling stability

**Fig. 4:**
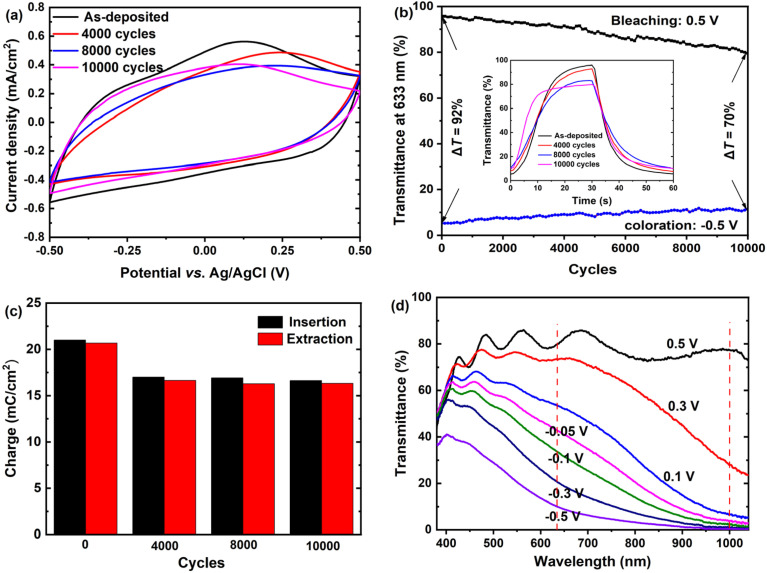
Long-term cycling stability of the amorphous WO_3_·2H_2_O film: (a) CV curves of the film recorded after various CA cycles (−0.5 V for 30 s and 0.5 V for 30 s) in a LiClO_4_/PC electrolyte; (b) variation of the transmittance at 633 nm for the film after different cycles, with the inset showing the corresponding variation in the response times; (c) inserted and extracted charge densities as a function of cycle number for the films after different cycles; (d) transmittance spectra of the film after 10 000 cycles measured under different applied potentials.

The long-term cycling stability of the amorphous WO_3_·2H_2_O film was evaluated by applying potential cycles between −0.5 V and 0.5 V in a 1.0 M LiClO_4_/PC solution. CV measurements were performed after various cycles to assess the electrochemical stability. As shown in Fig. 4(a), the CV shape remains largely unchanged, indicating that the film maintains its electrochemical stability over extensive cycles. However, a slight decrease in the CV area is observed as the number of cycles increases. Additionally, the redox peaks become less prominent overall, and even disappear, after 8000 cycles. However, after 10 000 cycles a tiny, broad peak reappears. These changes in the CV curves suggest that the film might undergo degradation or structural transformation after prolonged cycling. The transmittance at 633 nm was measured after different cycling intervals. As shown in Fig. 4(b), the film retains approximately 94%, 88% and 76% of its original optical modulation after 2000, 5000 and 10 000 cycles, respectively, demonstrating excellent electrochemical cycling stability. The reduced optical modulation leads to faster response times, with *t*_b_ decreasing from 17.5 s to 7.4 s and *t*_c_ decreasing from 18.1 s to 14.2 s after 10 000 cycles, as illustrated in the inset of Fig. 4(b). Fig. 4(c) presents the inserted and extracted charge densities as a function of cycle numbers for the films after different cycles. The initial charge density is 21.1 mC cm^−2^, which decreases to approximately 17.0 mC cm^−2^ after 4000 cycles and remains stable thereafter. The minor variations in charge density over the cycles imply high electrochemical stability. Furthermore, the nearly equal inserted and extracted charge densities across the cycles indicate excellent reversibility in the electrochromic processes. Fig. 4(d) demonstrates that even after 10 000 repetitive cycles, the film maintains its dual-band modulation behavior, achieving a maximal Δ*T* of 77% at 1100 nm and 70% at 633 nm.

**Fig. 5:**
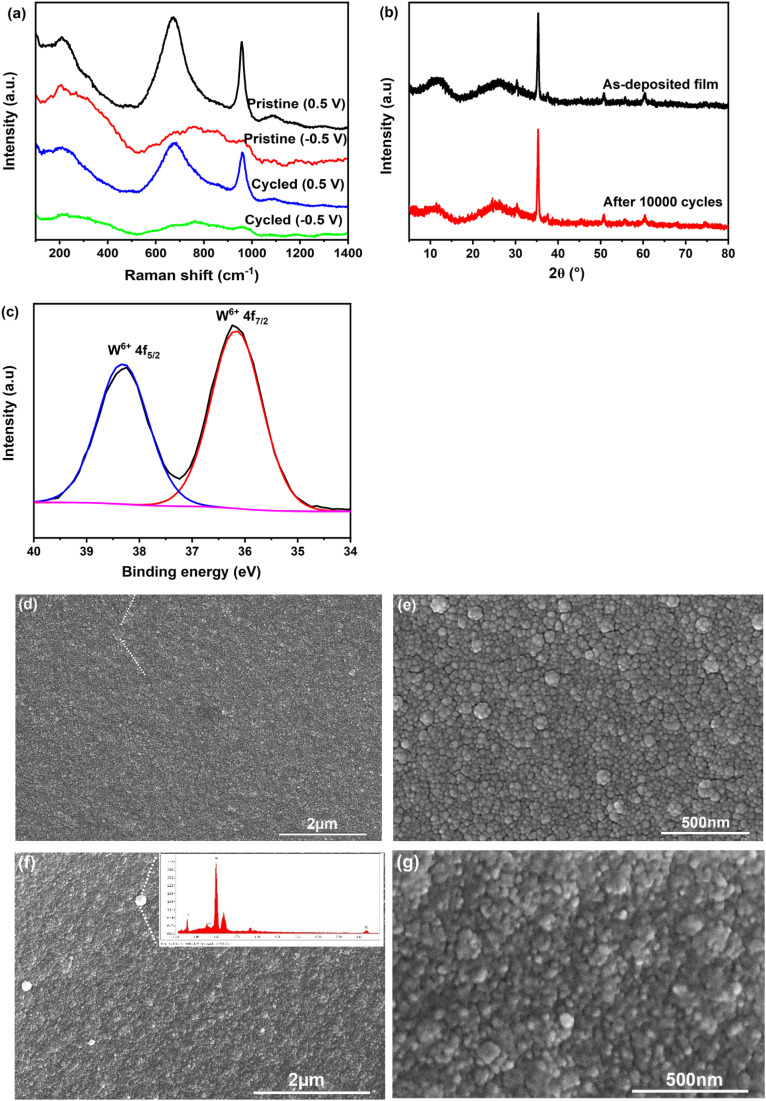
Composition and microstructural analyses of the amorphous WO_3_·2H_2_O film subjected to CA cycling (−0.5 V for 30 s and 0.5 V for 30 s) in LiClO_4_/PC electrolyte: (a) and (b) Raman spectra and XRD patterns of the as-deposited and the film after 10 000 cycles; (c) high-resolution W4f XPS spectrum of the film after 10 000 cycles; (d) and (e) SEM images of the film after 5000 cycles; (f) and (g) SEM images of the film after 10 000 cycles, with the inset of (f) showing the EDS result of the white small particles observed on the surface.

To gain insight into the reason behind the performance degradation of the amorphous WO_3_·2H_2_O film, we performed XRD, Raman and XPS analyses on the film after 10 000 cycles. As shown in Fig. 5(a) and (b), the XRD patterns and Raman spectra exhibit no significant changes, indicating that the layered structure of WO_3_·2H_2_O remains intact without any loss of interlayer water. This contrasts with previous studies that reported dehydration of WO_3_·2H_2_O into WO_3_·H_2_O in non-aqueous electrolytes.^31,32^ The high-resolution W 4f XPS spectrum of the cycled film in its bleached state (Fig. 5(b)) shows signals corresponding exclusively to W^6+^, with no detectable presence of tungsten in lower oxidation states. This suggests that Li^+^ ions are not trapped within the network, which is a critical factor for ensuring the electrochemical cycling stability of the film. These results are highly encouraging, as they demonstrate that the structure and composition of the film remain unchanged even after 10 000 cycles. This remarkable stability can be attributed to the stabilizing effect of water molecules within the interlayer space.

Furthermore, the surface morphology of the film was examined using SEM after different cycling intervals, as shown in Fig. 5(c–f). The film retains its initial surface morphology up to 5000 cycles. However, after 10 000 cycles, the film appears rougher, with grain boundaries becoming less distinct. This observation suggests the repeated insertion and extraction of Li^+^ ions induce surface dissolution or erosion. The observed morphological changes are likely responsible for reducing the available active sites for ion insertion and extraction, leading to a decline in the electrochemical activity of the film. This is supported by the weakening and eventual disappearance of the redox peaks in the CV curves after 8000 cycles. Furthermore, the less distinct grain boundaries hinder the efficient insertion of Li^+^ ions into the interlayer spacing, further diminishing the film's overall electrochemical capacity. Consequently, the optical modulation capability of the film gradually deteriorates over time. Upon close inspection, a small quantity of white particles was observed on the film surface. EDS analysis, as shown in the inset of Fig. 5(f), indicates that these particles are primarily composed of W and O, with an atomic ratio of O to W of approximately 2.7. It is therefore inferred that as the amorphous WO_3_·2H_2_O film dissolved, the tungsten species (likely in the form of tungstate anions, such as [WO_4_]^2−^) in the electrolyte could re-precipitate onto the film surface as WO_3_ or its reduced forms. The formation of these particles may introduce new redox-active sites, which could account for the reappearance of a tiny, broad redox peak in the CV curve after 10 000 cycles.

A brief comparison of the electrochromic performance of our amorphous WO_3_·2H_2_O film with other WO_3_ hydrate films is presented in ESI Table S1.† Our film demonstrates performance that is comparable to or superior to others, particularly in terms of optical modulation and cyclic stability. Notably, the film operates at an exceptionally low potential, a significant advantage for electrochromic devices as it reduces energy consumption and enhances material longevity. Compared to previously reported dual-band electrochromic crystalline WO_3_·H_2_O^13^ and amorphous WO_3_·0.9H_2_O,^7,9^ our WO_3_·2H_2_O film features a more open framework with larger interlayer spacing, which facilitates faster ion diffusion and higher ion storage capacity. Additionally, the presence of water molecules in the interlayer space provides enhanced flexibility during ion intercalation and de-intercalation processes, further improving the film's cyclic stability.

**Fig. 6:**
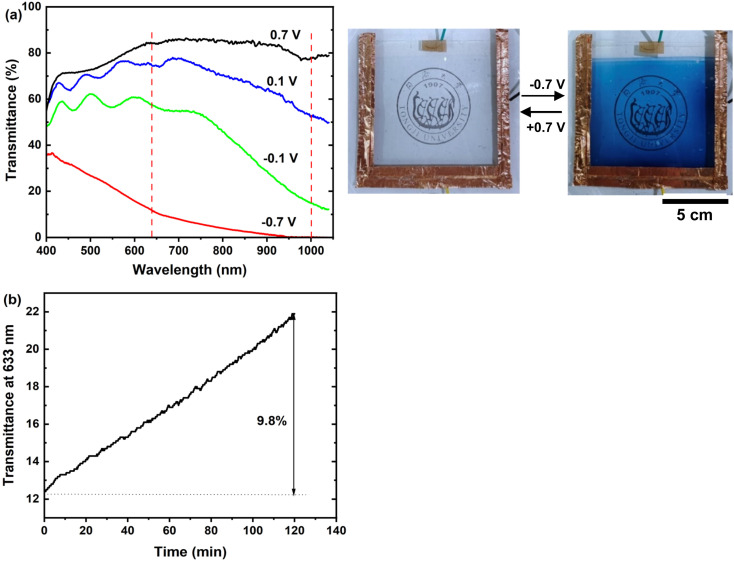
(a) Optical transmittance of a 10 cm × 10 cm device at various potentials, along with photographs of the device in its bleached state at 0.7 V and colored state at −0.7 V; (b) evolution of transmittance at 633 nm under open circuit voltage (OCV) after coloration at −0.7 V.

To evaluate the scalability of the electrodeposition method, we successfully deposited a large-area amorphous WO_3_·2H_2_O film on a 10 cm × 10 cm ITO glass slide under the same conditions as those used for small-area films. A 100 cm^2^ prototype device was then fabricated following the method reported by Fang *et al.*,^33^ using bare ITO glass as the counter electrode, WO_3_·2H_2_O film on ITO as the working electrode, and polyacrylamide (PAAm) hydrogels containing LiCl as the gel electrolyte. As illustrated in Fig. 6(a), the device exhibits robust modulation in both the visible and NIR ranges, achieving significant optical modulation of approximately 70% at 633 nm and 78% at 1100 nm. Under an applied potential of −0.7 V, the device displays uniform coloration. The measured response times for coloration and bleaching are less than 40 s and 30 s, respectively. These response times are reasonable given the large area of the electrochromic film and relatively lower ionic conductivity of the gel electrolyte compared to liquid electrolytes. Additionally, the optical memory effect of the device was evaluated after coloration at −0.7 V. The evolution of transmittance at 633 nm under open circuit voltage (OCV) is depicted in Fig. 6(b). Over a period of 120 min, the transmittance of the device increases by 9.8%. This relatively weak color persistence, compared to anhydrous WO_3_,^34,35^ can be attributed to the shallow “trapping” of Li^+^ ions within the open structure of WO_3_·2H_2_O. The structural feature allows for easier ion extraction, contributing to faster self-bleaching.

## Conclusions

In this study, we successfully fabricated amorphous WO_3_·2H_2_O films through cathodic electrodeposition using a precursor solution primarily composed of monomeric diperoxotungstate. The precursor solution was prepared by dissolving a powder with the composition [WO_2_(O_2_)H_2_O]·1.66H_2_O, synthesized *via* the reaction of tungsten powder with H_2_O_2_. The resulting films demonstrated remarkable electrochromic performance, including high optical modulation in both visible and NIR regions (∼92% at 633 nm and ∼86% at 1100 nm), fast response times (17.5 s for bleaching and 18.1 s for coloration at 633 nm; 5.0 s for bleaching and 7.1 s for coloration at 1100 nm), and high coloration efficiencies (204.2 cm^2^ per C at 1100 nm and 72.3 cm^2^ per C at 633 nm). Notably, the films exhibited exceptional cyclic stability, retaining 94% of their initial optical modulation after 2000 cycles and 76% after 10 000 cycles at 633 nm, representing a significant improvement over previously reported WO_3_ films. These superior properties highlight the critical role of interlayer water in enhancing ion diffusion kinetics and overall film stability. Additionally, the scalability of the electrodeposition method was demonstrated by the successful fabrication of large-area films (10 cm × 10 cm) and electrochromic devices. The combination of scalability, cost-effectiveness, and simplicity makes this approach a promising strategy for producing high-performance WO_3_ films, with potential applications in energy-efficient smart windows.

## Data availability

The data supporting the findings of this study were collected during the completion of a master dissertation and are currently stored on a personal computer. Due to data protection, they are not available in a public repository. However, the data may be accessed upon reasonable request. Contact Aihua Yao *via* email to 07182@tongji.edu.cn.

## Author contributions

All authors contributed to the investigation, methodology, writing – original draft, writing – review, formal analysis and editing.

## Conflicts of interest

The authors have no conflicts of interest to declare with respect to the research, authorship and publication of this article.

## Supplementary Material

RA-015-D4RA08851D-s001
