# Supplementary material for: Cathodic electrodeposition of amorphous tungsten oxide dihydrate film for dual-band electrochromic modulation
Source: RSC Adv. 2025 Feb 18;15(7):5242–51. doi: 10.1039/d4ra08851d (PMC11833444; doi:10.1039/d4ra08851d)
Supplement: RA-015-D4RA08851D-s001 [file RA-015-D4RA08851D-s001.pdf]

Supplementary Information (SI) for RSC Advances.
This journal is © The Royal Society of Chemistry 2025

Supplementary Information

Table S1 Performance parameters of the amorphous $WO_3·2H_2O$ film compared with those of WO_3 hydrate films reported in the literature

Material	ΔT	Operation potential (V)	t_c/t_b (s)	*CE* (cm^2/C)	Cycling stability
Crystalline $WO_3·H_2O$ Film [1]	59% at 1200 nm, 13% at 550 nm	1.6~4.0 (vs. Li/Li^+)	-	96.2 at 1200 nm, 20.7 at 550 nm	-
Crystalline $WO_3·2H_2O$ nanoplate film [2]	90.4% at 1600 nm	-0.2~0.4 (vs. Ag/AgCl)	-	322.6 at 1600 nm	Retaining 93.7% of ΔT after 500 cycles
Amorphous $WO_3·0.9H_2O$ film [3]	69% at 1100 nm, 24% at 550 nm	-0.8~0.8 (*vs.* Ag/AgCl)	2.3/2.1 at 1100 nm	312 at 1100 nm	Retaining 97% of ΔT after 12000 cycles at 1100 nm; retaining 87% after 1000 cycles at 550 nm
Amorphous and crystalline Ti-doped $WO_3·2H_2O$ film [4]	83.8% at 633 nm, 72.5% at 1050 nm	-0.5~0.5 (*vs.* Ag/AgCl)	15.3/14.9 at 633 nm, 3.5/16.0 at 1050 nm	22.8 at 633 nm, 90.8 at 1050 nm	Retaining 52.3%% of ΔT at 633 nm and 26% at 1050 nm after 500 cycles
Crystalline $WO_3·H_2O$ nanoplates film [5]	58% at 633 nm	-1~-3 (*vs.* Ag/AgCl)	9.6/5.1	38.2	-
$WO_2(O_2)H_2O·1.66H_2O$ nanocrystal film [6]	32% at 632 nm	-1.5~0.5 (*vs.* Ag/AgCl)	7.8/1.7	5.74	-
$3WO_3·H_2O$ nanoplate film [7]	38% at 633nm	-0.3~0.3 (*vs.* Ag/AgCl)	4.3/1.4	112.7	-
Amorphous $WO_3·2H_2O$ film (this work)	92% at 633 nm and 86% at 1100 nm	-0.5~0.5 (*vs.* Ag/AgCl)	18.1/17.5 at 633 nm, 7.1/5.0 at 1100 nm	204.2 cm^2/C at 1100 nm, 72.3 cm^2/C at 633 nm	retaining 94% of ΔT after 2000 cycles and 76% after 10000 cycles at 633 nm; retaining 18% of ΔT after 10000 cycles at 1100 nm.

[1] J. Fortunato, B.Z. Zydlewski, M. Lei, N.P. Holzapfel. M. Chagnot, J.B. Mitchell, H. Lu, D. Jiang, D.J. Milliron, V. Augustyn. Dual-band electrochromism in hydrous tungsten oxide, ACS Photonics, 10 (2023) 3409-3418.

[2] Z. Wang, W. Gong, X. Wang, Z. Chen, X. Chen, J. Chen, H. Sun, G. Song, S. Cong, F. Geng, Z. Zhao. Remarkable near-infrared electrochromism in tungsten oxide driven by interlayer water-induced battery-to-pesedocapacitor transition. ACS Appl. Mater. Interfaces, 12 (2020) 33917-33925.

[3] W. Zhao, J. Wang, B. Tam, H. Zhang, F. Li, A. Du, W. Cheng. Structural water in amorphous tungsten oxide hydrate enables fast nad ultrastable regulation of near-infrared light transmittance, Adv. Opt, Mater., 11 (2023) 2202774.

[4] X. Sun, D. Wang, W. Wu, X. Zhao, Z. Zhang, B. Wang, X. Rong, G. Wu, X. Wang. Amorphous and crystalline Ti-doped $WO_3 \cdot 2H_2O$ for dual-band electrochromic smart windows, ACS Sustain. Chem. Eng., 12 (2024) 5459-5467.

[5] C.Y. Ng, K.A. Razak, Z. Lockman. Effect of annealing on acid-treated $WO_3 \cdot H_2O$ nanoplates and their electrochromic properties, Electrochim. Acta, 178 (2015) 673-681.

[6] S. Wang, K. Dou, Y. Zou, Y. Dong, J. Li, D. Ju, H. Zeng. Assembling tungsten oxide hydrate nanocrystal colloids formed by laser ablation in liquid into fast-response electrochromic films, J. Colloid Interf. Sci., 489 (2017) 85-91.

[7] Z. Jiao, X. Wang, J. Wang, L. Ke, H.V. Kemir, T. W. Koh, X. Sun. Efficient synthesis of plate-like crystalline hydrated tungsten trioxide thin films with highly improved electrochromic performance, Chem. Commun., 48 (2012) 365-367.
